# Trend in antibiotic prescription to children aged 0–6 years old in the capital region of Denmark between 2009 and 2018: Differences between municipalities and association with socioeconomic composition

**DOI:** 10.1080/13814788.2021.1965121

**Published:** 2021-09-06

**Authors:** Sif Binder Larsen, Maria Louise Veimer Jensen, Lars Bjerrum, Volkert Siersma, Christine Winther Bang, Jette Nygaard Jensen

**Affiliations:** a Department of Public Health, Section of General Practice and Research Unit for General Practice, University of Copenhagen, Copenhagen, Denmark; b Department of Clinical Microbiology, Herlev and Gentofte Hospital, University of Copenhagen, Herlev, Denmark

**Keywords:** Antibiotics, children, primary health care, socioeconomic status, infectious diseases

## Abstract

**Background:**

To curb future antibiotic resistance it is important to monitor and investigate current prescription patterns of antibiotics.

**Objectives:**

To examine trends in antibiotic prescription to children aged 0–6 years old and the association with socioeconomic status of municipalities in the Capital region of Denmark between 2009 and 2018.

**Methods:**

This is a register-based study combining data on antibiotic treatments from 2009 to 2018, inhabitant-data and socioeconomic municipality scores. Subjects were children aged 0–6 years, residing in the Capital Region of Denmark. The study quantifies the use of antibiotics as number of antibiotic treatments/1000 inhabitants/year (TIY), inhabitants defined as children aged 0–6. Socioeconomic status of the municipalities is evaluated by a score from 3 to 12.

**Results:**

The average TIY of the municipalities decreased from 741.2 [95%CI 689.3–793.2] in 2009 to 348.9 [329.4–368.4] in 2018. The difference between the highest and lowest prescribing municipalities was reduced from 648.3 TIY in 2009–212.5 TIY in 2018. The average increase in TIY per unit increase in socioeconomic municipality score changed from 20.05 [7.69–31.06] in 2009 to −4.58 [-16.02–5.60] in 2018, representing a decreasing association between socioeconomic municipality score and use of antibiotic in the respective municipalities.

**Conclusion:**

The trend in antibiotic prescription to children aged 0–6 years old decreased substantially in all the investigated municipalities in the 10-year study period. Local differences in prescription rates declined towards a more uniform prescription pattern across municipalities and association with socioeconomic status of the municipalities was reduced.

KEY MESSAGES

- Antibiotic prescriptions for children have been reduced by 46.3% from 2009 to 2018
- Local variation in antibiotic prescriptions between municipalities are becoming more uniform
- An association between prescription rates and socioeconomic status was found by 2009 but declined towards disappearing by 2018

## Introduction

Antimicrobial resistance (AMR) is one of the biggest threats to global health, and both national and international agencies have published action plans to curb the rising resistance [1,2]. The correlation between AMR and the use of antibiotics is well established [3,4]. It is of great importance to identify and prevent unnecessary use of antibiotics, to avoid a worst-case scenario of returning to circumstances of the pre-antibiotic era [5].

A group that has been in focus for receiving unnecessary antibiotics is children of the pre-school age. As far back as 1975, it has been described that a major part of prescribed antibiotics for young children is based on indications such as upper respiratory tract infections, where antibiotics have been proven to have either limited or no effect [6–8]. This age group has the highest prescribing rate of antibiotics among children, and the prescribing rate has been increasing since the problem was first acknowledged [9,10].

Another aspect of antibiotic prescription patterns is that there is a considerable geographical variation both between [11] and within countries [12,13]. The within-country variation is pronounced among children [14–16]. Socioeconomic factors such as education level, income and occupational status of the parents have been associated with geographical variation in use of antibiotics for children [14,17,18].

In Denmark, the primary healthcare sector prescribes 90% of all antibiotics for human use [10,19]. The Capital Region of Denmark has the highest rate of antibiotic prescriptions among children aged 0–1 years, and the second-highest among children aged 2–4 years, compared to other regions of Denmark [9]. A decrease in antibiotic consumption has been registered during the last 10-years for almost all age groups (except >80 years old), but most noticeable in the 0–4 years age group [10]. It has not yet been investigated if there are local differences in this development. Knowledge of antibiotic prescription patterns and development of these may give leads to which interventions may be useful to reduce unnecessary use of antibiotics.

This study examines the trend in antibiotic prescription to children aged 0–6 years old between municipalities in the Capital Region of Denmark from 2009 to 2018 and to investigate the association between reduction of antibiotic prescriptions and socioeconomic composition of municipalities.

## Methods

### Study design

This is a register-based study combining data on antibiotic treatments from the past ten years, inhabitant-data from Statistics Denmark and socioeconomic municipality scores by the Capital Region of Denmark. The study population comprised children aged 0–6 years, living in the Capital Region of Denmark in each calendar year of the study. The Capital Region is one of five regions in Denmark, with 1,795,868 inhabitants in 2018. The region is divided in 29 municipalities, with the largest being Copenhagen with 622,698 inhabitants and the smallest Dragoer with 14,279 inhabitants [20]. The municipality of Bornholm was not included in this study, as its position as an isolated island does not represent the urban environment of the rest of municipalities in the analysis. When we refer to the municipalities in the Capital Region, we refer to the 28 municipalities located on Zealand.

The socioeconomic status of the municipalities differs. The wealthiest municipalities are characterised by a population with long educations, high income and high adherence to guidelines of healthy eating and physical activity. The most deprived municipalities are characterised by a population with a lower affiliation with the labour market, a high number of non-western inhabitants and a larger share of smokers [21].

### Outcomes

The study population was a dynamic cohort of children aged 0–6 years living in municipalities in the Capital Region of Denmark from 2009 to 2018. Data on the number of children aged 0–6 years living in the municipalities was extracted from Statistics Denmark [22]. This governmental institution collects and maintains electronic registers for a broad spectrum of statistical and scientific purposes.

All antibiotics prescribed in the primary healthcare sector from 1 January 2009 to 31 December, 2018 for children aged 0–6, were extracted from a larger dataset from a central pharmacy settlement system within the Danish Regions. Data was on aggregated level, meaning the number of antibiotic prescriptions is connected to the age group instead of the individual child. Each prescription registered corresponds to a redeemed treatment. Antibiotic prescriptions from out-of-hour services are not included.

To make sure the numbers of antibiotics treatments are comparable between municipalities, this study quantified the use of antibiotics as number of antibiotic treatments/1000 inhabitants/year (TIY), inhabitants defined as children aged 0–6.

Data on socioeconomic municipality score was extracted from the reports *Health profile of the Capital Region and municipalities* from 2008, 2010, 2013, and 2017 [21,23–25] written by the Capital Region of Denmark. The reports are based on all inhabitants aged 25 years and older, which in the first report (2008) corresponds to 1,173,500 inhabitants. In the reports, a socioeconomic score for each municipality was created based on (1) educational level (2) employment status and (3) average income.

For each of these three variables, the municipalities were given a score of one to four points, resulting in a total score from 3 to 12. Municipalities with the most advantageous socioeconomic profile have the least points.

### Analysis

The association between socioeconomic score and antibiotic TIY of the municipalities for each of the years in the 10-year period were assessed by a separate linear effect of socioeconomic score for each year in linear mixed model regression analyses, using a municipality random effect to control for the excess correlation between TIY assessments of the same municipality at different years. These associations are presented with 95% confidence intervals. Results are shown as both untransformed TIY data and logarithmic transformed TIY data. The latter gives multiplicative assessments of the associations and stabilises the variance.

Maps visualising differences between the municipalities were made using ArcMap (a part of ArcGIS Desktop 10.6.1), a GIS mapping software by Esri. Statistical analyses were performed using SPSS version 22, and R version 3.6.0.

## Results

In the 10-year period, a total of 779,129 antibiotic treatments were redeemed, of which 64.8% were redeemed in the first five years (2009–2013). In 2009, 139,259 children aged 0–6 years lived in the Capital Region of Denmark and received an average of 694.9 TIY. In 2018, the number of 0–6-year-old children increased to 141,240 children, who received an average of 321.6 TIY.

### Local variation in decrease

The average TIY of the municipalities was 741.2 95%CI [689.3–793.2] in 2009, which decreased to 348.9 [329.4–368.4] TIY in 2018. The socioeconomic municipality score remained stable in the 10-year period, with only one municipality changing score with more than one point. Characteristics of demographics and antibiotic treatments for the 28 municipalities are shown in Supplementary Material S1.

**Figure 1.**
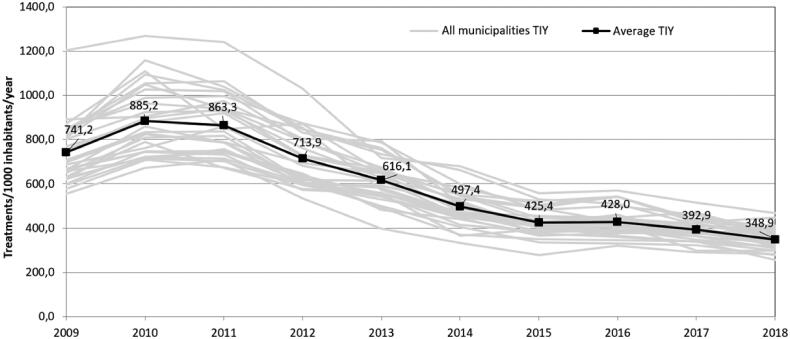
Development of TIY in the municipalities (grey lines) and the average TIY of the municipalities (black line) from 2009 to 2018.

A general trend across municipalities of decreasing TIY during the 10-year period was observed (Figure 1). The municipalities average reduction in TIY from 2009–2018 was 52.4% [50.2–54.6, range 41.7–62.5].

The municipality of Ishoej had the highest prescription rate throughout the 10-year period. In 2009 a total of 1 202.4 TIY were prescribed, in 2018 the highest rate declined to 467.9 TIY. The municipality using least antibiotics in 2009 was Alleroed with 554.1 TIY, in 2018 the lowest number of treatments was recorded in the municipality of Egedal with 255.4 TIY. Local variation is visualised in Supplementary Material S2.

In 2009, the differences between the municipalities with the highest and lowest TIY were considerable, with a gap of 648.3 TIY between Ishoej and Alleroed. This gap decreased with two thirds during to 10-year period, to 212.5 TIY between Ishoej and Egedal.

### Socioeconomic municipality score

The analysis of the association between socioeconomic municipality score and use of antibiotics in the 10-year period, showed a trend of significant association at the beginning of the period that disappeared towards the end of the period (Table 1). The analysis showed an increase in mean antibiotic TIY by 20.05 [7.69–31.06] in 2009 for each point the municipalities increases in socioeconomic score (i.e. socioeconomic status gets more disadvantageous). This changed to −4.58 [−16.02 to 5.60] in 2018, now predicting a mean decrease in antibiotic TIY as the socioeconomic municipality score increases. This tendency was consistent with the multiplicative associations, where in 2009 each point increase in socioeconomic score raised the mean antibiotic TIY by 2.6% [0.9–4.1], which decreased to 0.1% [−1.4 to 1.5] in 2018.

**Table 1.** Association between antibiotic TIY (treatments/1000 inhabitants/year) and socioeconomic municipality score.

	Increase in mean antibiotic TIY per point increase in socioeconomic municipality score*		Multiplicative increase in mean antibiotic TIY per point increase in socioeconomic municipality score**	
Year	Increase	95% CI	Factor	95% CI
2009	20.05	[7.69–31.06]	1.026	[1.009–1.041]
2010	12.93	[0.57–23.94]	1.012	[0.995–1.028]
2011	19.32	[6.96–30.34]	1.020	[1.003–1.035]
2012	13.45	[1.09–24.46]	1.018	[1.002–1.034]
2013	11.33	[−0.10–21.50]	1.021	[1.005–1.035]
2014	5.42	[−6.00–15.60]	1.017	[1.001–1.031]
2015	0.38	[−11.05–10.55]	1.010	[0.995–1.025]
2016	0.45	[−10.98–10.62]	1.010	[0.995–1.024]
2017	−6.96	[−18.40–3.22]	0.993	[0.978–1.007]
2018	−4.58	[−16.02–5.60]	1.001	[0.986–1.015]

*F-test for homogeneity of effect over time *p* < 0.0001.

**F-test for homogeneity of effect over time *p* < 0.0001.

## Discussion

### Main findings

This study demonstrated that during the 10-year timespan from 2009 to 2018, there has been a considerable reduction in use of antibiotics among children aged 0–6 years. All municipalities in the Capital Region of Denmark reduced the number of antibiotic treatments by 40–60% for this age group. The difference between the highest and lowest prescription rate declined with two thirds during the 10-year period, indicating a more uniform prescription pattern between the municipalities. We found a declining association between socioeconomic municipality scores and antibiotic treatments from 2009 towards 2018, indicating a reduced association of socioeconomic status with antibiotic treatment patterns.

### Strengths and limitations

This study contains strengths and limitations that need to be addressed. A strength of our study is the availability of high-quality data from Statistics Denmark and Danish National Prescription Database [22,26]. The data, therefore, represents a realistic quantification of antibiotic treatments prescribed in the primary healthcare sector from 2009–2018.

This study focussed on redeemed prescriptions within the primary care sector; hence we have no knowledge of actual compliance. However, compliance may differ among different social classes and should be studied further.

Data included in this study was based on information about antibiotic treatments on an aggregated level, therefore, we were not able to distinguish between children receiving few or several prescriptions. In addition, it was not possible to control for potential confounders on an individual level, such as gender, siblings and birth weight, which have been shown to affect the risk of infections [18,27].

The socioeconomic municipality score was based on the report *Health profile of the Capital Region and municipalities*, which is published every 2nd to 4th year [28]. The socioeconomic municipality score remained stable during the 10-year period. Only one municipality differed >1 point, 12 municipalities increased their score by one point and two municipalities scored one point less in 2017 compared to 2008 [21,23].

The socioeconomic score was based on three parameters; income, education and employment status. In the reports from 2008 and 2017 [21,23], the educational status was classified slightly different. In 2008, it was defined as the percentage of inhabitants with a maximum of 10 years education and in 2017 as the percentage with a maximum of 12 years of education. We do not believe that this slight difference in educational classification status influenced the overall socioeconomic municipality score based on income, education and employment status.

Various variables that may be confounder candidates for the association between socioeconomic status and infection rates, and thereby the use of antibiotics, may rather be viewed as aspects of or caused by the socioeconomic status, e.g. birth weight [29]. If we control for these intermediate variables, we end up removing some of the association between socioeconomic status and the use of antibiotics. A safe interpretation steers away from causal statements and states that there is an association. The causes of this association are to be found in variables related to the socioeconomic status concept and are not determined in the present analysis.

### Results in relation to other studies

Our study’s observed decrease in antibiotic treatments is in line with data from national report on antibiotic use (DANMAP) [10].

Many studies have investigated the association between variations in antibiotic use and socioeconomic factors but the findings disagree. A between-country variation on the impact of socioeconomic status of parents on antibiotic prescription patterns for children has been found [30]. Studies by Thrane et al. (2003) and Jensen et al. (2016), who investigated the correlation between socioeconomic status of parents and use of antibiotics for children in Denmark, found that a high educational level of parents was associated with a lower number of antibiotic treatments [17,18]. A study in Germany by Koller et al. (2012), who analysed area deprivation in relation to antibiotic prescribing, found that children in the most deprived areas had a 20% higher chance of receiving an antibiotic treatment compared to children in the least deprived areas [14]. Covvey et al. (2014) analysed socioeconomic deprivation in relation to antibiotic prescriptions in Scotland for all age groups from 2010 to 2012. They found a significant association of socioeconomic deprivation on prescription rates, but no association between socioeconomic status and the development in treatments during the 3-year period, where a total increase in antibiotic prescriptions was found [31].

Saust et al. (2018) measured quality of antibiotic prescribing in general practice, and found overuse of antibiotics for respiratory tract infections [32]. This finding is in line with those of Lous et al. (2019), who found that 41% of children aged 0–5 received an respiratory tract infection diagnosis in an out-of-office setting and 12% was diagnosed with acute otitis media, for which 70% received an antibiotic prescription, despite the proven lack of effect [6,33]. In relation to our results, this raises the question if part of the decline in antibiotic prescriptions in general practice is due to this increased use of out-of-hour care described by Lous et al. (2019).

### Efforts and implications

Different efforts have been set out to limit the use of antibiotics and thereby curb the rising AMR in Denmark. In 2007, a new 7-valent conjugated pneumococcal vaccine was introduced in the Danish Childhood Vaccination programme [34]. In 2012, the Danish National Health and Medicine Authority introduced a set of national guidelines on antibiotic prescribing, aiming to make prescription of antibiotics more rational and reduce excessive use [35]. Besides actions on reducing infection frequency and unnecessary use of antibiotics, there have been efforts to raise awareness about antibiotics and the consequences of unnecessary use with an ongoing informational campaign ‘*Antibiotika eller ej’* (Antibiotics or not), addressing both medical professionals and the general population - especially parents [36]. We believe these efforts might have resulted in a more uniform prescription- and demand pattern for antibiotics among general practitioners and parents equalising the influence of socioeconomic status.

According to the National action plan on antibiotics [2] the goal is to reduce antibiotics in the primary healthcare sector below 350 TIY. Our data shows that for the age group of 0–6 years old, the numbers are approaching this with 16 of 28 municipalities meeting this goal in 2018.

Our study investigated the correlation between a decrease in antibiotic consumption and socioeconomic status, which to our knowledge has not been done before. Knowledge of prescription patterns and driving factors of these is important to target efforts to reduce the overuse of antibiotics. The mechanisms behind the notable change in prescription patterns found in our study are yet to be understood and further research is needed to fully understand what has led to this significant reduction in use of antibiotics. The drivers of the association between socioeconomic status and use of antibiotics are not determined and would benefit of more elaborative research. Furthermore, the trend towards a more uniform prescription pattern shows a need for a more generalised effort contrary to 10 years ago, where a focus towards the socioeconomic deprived municipalities would have been advantageous.

## Conclusion

Our study shows that the seemingly increase in antibiotic prescription to children aged 0–6 years old has turned and additionally been subject to a substantial reduction in the 10-year timespan. The local differences in prescription rates have declined towards a more uniform prescription pattern across municipalities. The association between socioeconomic status of the municipality and the prescription of antibiotics declined towards disappearing by 2018.

## Supplementary Material

Supplemental Material
