# Supplementary material for: Trend in antibiotic prescription to children aged 0–6 years old in the capital region of Denmark between 2009 and 2018: Differences between municipalities and association with socioeconomic composition
Source: Eur J Gen Pract. 2021 Sep 6;27(1):257–63. doi: 10.1080/13814788.2021.1965121 (PMC8425679; doi:10.1080/13814788.2021.1965121)
Supplement: Supplemental Material [file IGEN_A_1965121_SM4000.docx]

| Supplement material S1. Characteristics of the 28 municipalities of the Capital Region of Denmark | | | | | | | | | | |
| --- | --- | --- | --- | --- | --- | --- | --- | --- | --- | --- |
| Municipality  (map reference) | No. of children  0-6 years | | Total no. of inhabitants | Treatments/1000 inhabitants/year (TIY)* | | | Absolute change in TIY | Relative change in TIY (%)** | Socio-economic municipality group*** | |
|  | 2009 | 2018 | 2018 | 2009 | 2018 | 2009-2018 | | 2009-2018 | | 2017 |
| Allerød (ALL) | 2256 | 1992 | 25544 | 554,1 | 281,6 | | 272,5 | 49,2 | | 3 |
| Gentofte (GEN) | 6066 | 5494 | 75265 | 620,3 | 328,5 | | 291,8 | 47,0 | | 3 |
| Hørsholm (HØR) | 1795 | 1589 | 25091 | 632,3 | 346,1 | | 286,2 | 45,3 | | 3 |
| Lyngby-Taarbæk (LYN) | 4134 | 4103 | 55850 | 648,5 | 308,8 | | 339,7 | 52,4 | | 3 |
| Rudersdal (RUD) | 4497 | 4156 | 56545 | 594,8 | 322,9 | | 271,9 | 45,7 | | 3 |
| Dragør (DRA) | 1122 | 1137 | 14279 | 812,8 | 376,4 | | 436,4 | 53,7 | | 4 |
| Furesø (FUR) | 3370 | 3327 | 40924 | 600,0 | 316,2 | | 283,8 | 47,3 | | 4 |
| Egedal (EGE) | 4128 | 3395 | 43267 | 622,8 | 255,4 | | 367,4 | 59,0 | | 5 |
| Frederiksberg (FBR) | 8187 | 8370 | 104735 | 656,3 | 335,5 | | 320,8 | 48,9 | | 5 |
| Vallensbæk (VAL) | 1336 | 1581 | 16642 | 872,0 | 396,0 | | 476,1 | 54,6 | | 5 |
| Fredensborg (FBO) | 3320 | 2952 | 40764 | 662,0 | 331,3 | | 330,7 | 50,0 | | 6 |
| Gladsaxe (GLA) | 5679 | 5795 | 69624 | 750,5 | 384,8 | | 365,7 | 48,7 | | 6 |
| Hillerød (HIL) | 4394 | 3796 | 50874 | 663,4 | 339,8 | | 323,6 | 48,8 | | 6 |
| Frederikssund (FRS) | 3744 | 2901 | 45373 | 688,0 | 404,0 | | 284,0 | 41,3 | | 7 |
| Gribskov (GRI) | 3091 | 2291 | 41280 | 696,9 | 395,9 | | 301,0 | 43,2 | | 8 |
| Helsingør (HEL) | 4997 | 3926 | 62661 | 818,1 | 409,6 | | 408,5 | 49,9 | | 8 |
| København (KØB) | 44566 | 51774 | 622698 | 628,0 | 277,6 | | 350,5 | 55,8 | | 8 |
| Ballerup (BAL) | 3927 | 3745 | 48377 | 852,8 | 368,5 | | 484,3 | 56,8 | | 9 |
| Glostrup (GLO) | 1748 | 1821 | 22533 | 843,2 | 448,1 | | 395,1 | 46,9 | | 9 |
| Herlev (HER) | 2173 | 2479 | 28625 | 705,9 | 298,5 | | 407,4 | 57,7 | | 9 |
| Rødovre (RØD) | 3065 | 3430 | 39887 | 801,0 | 326,5 | | 474,4 | 59,2 | | 9 |
| Tårnby (TÅR) | 3444 | 3535 | 43033 | 844,4 | 316,8 | | 527,5 | 62,5 | | 9 |
| Hvidovre (HVI) | 4168 | 4586 | 53498 | 769,9 | 332,3 | | 437,6 | 56,8 | | 10 |
| Halsnæs (HAL) | 2528 | 1881 | 31378 | 670,5 | 298,8 | | 371,7 | 55,4 | | 11 |
| Høje-Taastrup (HØJ) | 4501 | 4030 | 50718 | 843,6 | 363,8 | | 479,8 | 56,9 | | 11 |
| Albertslund (ALB) | 2421 | 2167 | 27859 | 892,2 | 374,7 | | 517,5 | 58,0 | | 12 |
| Brøndby (BRØ) | 2734 | 2965 | 35555 | 808,0 | 362,6 | | 445,4 | 55,1 | | 12 |
| Ishøj (ISH) | 1868 | 2022 | 22989 | 1202,4 | 467,9 | | 734,5 | 61,1 | | 12 |
| Total | 139259 | 141240 | 1795868 | 741.2^#^ | 348.9^#^ | | 392.4^#^ | 52.4^#^ | | - |
| *Total amount of antibiotic treatments prescribed for 0-6 years old in the different municipalities in 2009 and 2018 respectively divided with total number of children living in the municipalities multiplied with 1000.  **Difference from 2009-2018 divided with treatments in 2009 in percent.  ***Data only shown from 2017 as they remain steady throughout the 10-year period.  # Average of municipalities | | | | | | | | | | |

**
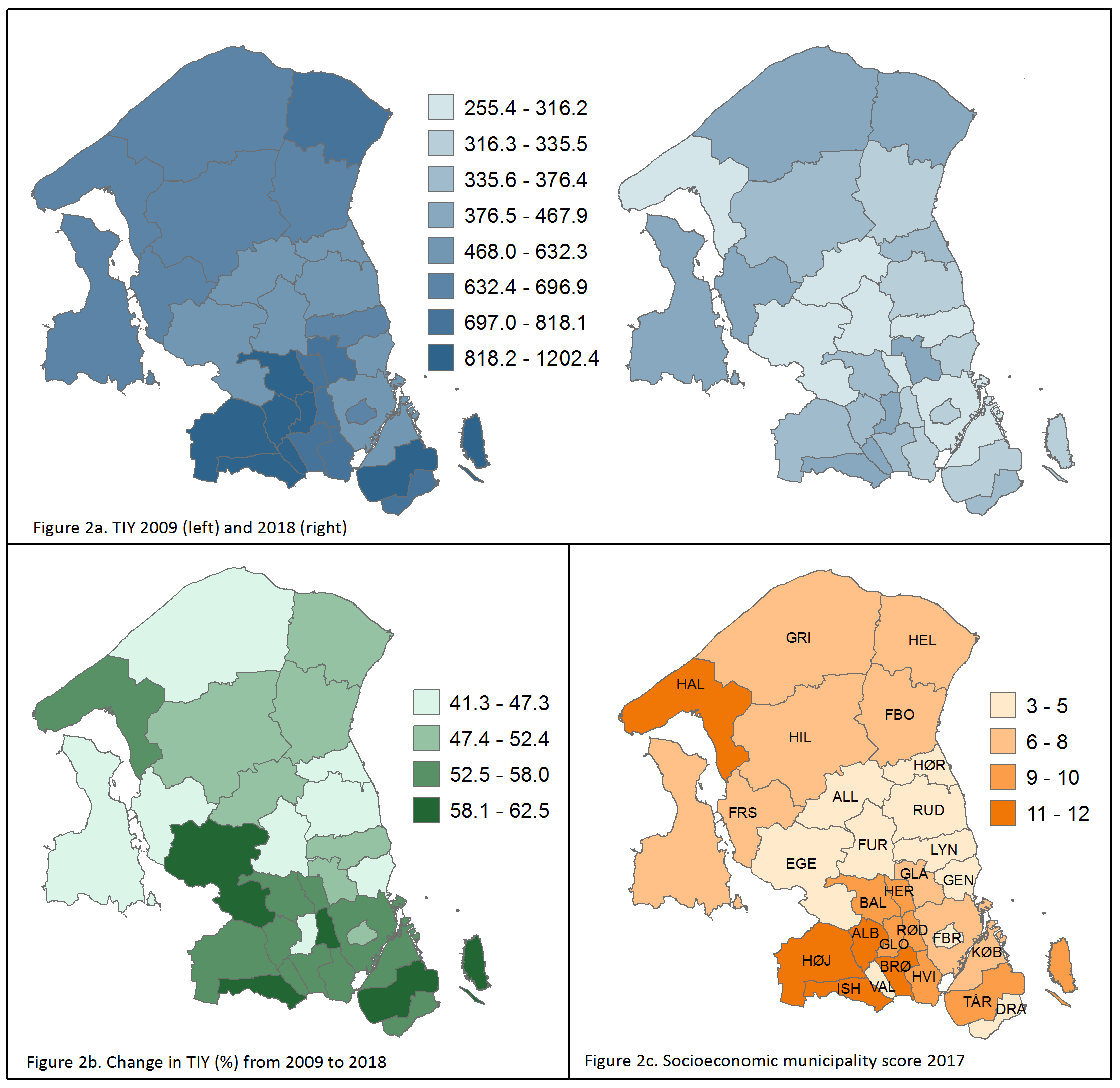
**

**Supplement material S2**. Absolute TIY in 2009 and 2018 (2a), relative reduction in TIY (%) from 2009 to 2018 (2b) and geographical variation in socioeconomic municipality score (2c). Results on percentage change and socioeconomic municipality score are grouped in quartiles.
